# Study of genes associated with the ‘anger-in’ and ‘anger-out’ emotions of humans using a rat model

**DOI:** 10.3892/etm.2015.2246

**Published:** 2015-02-02

**Authors:** YINGHUI GUO, HUIYUN ZHANG, JIE GAO, SHENG WEI, CHUNHONG SONG, PENG SUN, MINGQI QIAO

**Affiliations:** 1 College of Basic Medical Sciences, Shandong University of Traditional Chinese Medicine, Jinan, Shandong 250355, P.R. China; 2 Key Laboratory of Classic Theories on Traditional Chinese Medicine, Ministry of Education, Shandong University of Traditional Chinese Medicine, Jinan, Shandong 250355, P.R. China

**Keywords:** anger-in, anger-out, emotions

## Abstract

The aim of the present study was to investigate the genes associated with ‘anger-in’ (tendency to suppress anger) and ‘anger-out’ (tendency to express anger through verbal or physical means) emotions in humans. Wistar rats were divided into five groups (n=10/group), based on the type of model and the Chinese medicinal formulation administered, and the rat models were established. The five groups were as follows: Normal control (control), anger-in model (AIM), anger-in Jingqianshu-administered (AIA), anger-out model (AOM) and anger-out Jingqianping-administered (AOA). Open-field, resident-intruder and aggressive behavior tests were carried out, as well as gene expression analysis, reverse transcription-quantitative polymerase chain reaction and western blot analyses. The body weights of the rats in the AIM and AOM groups were significantly lower than those of the control group rats. The open-field test indicated that the scores in the AOM group were significantly higher (P<0.05) than those in the AIM group. The aggression scores of the rats in the AOM group were significantly higher than those of the AIM group rats. Jingqianshu and Jingqianping granules attenuated the behavioral changes of the rats. *5*-*Htr2C*, *GABA_B_R2* and *5-Htr3B* were associated with anger-in and anger-out emotions. Jingqianping and Jingqianshu granules attenuated the changes in the mRNA expression of *5-Htr2C*, *GABA_B_R2* and *5-Htr3B*, as indicated by RT-qPCR, and showed similar effects on protein expression, as demonstrated by western blot analysis. The present study demonstrated that the anger-in and anger-out emotions of rats are closely associated with *5-Htr2C, GABA_B_R2* and *5-Htr3B* genes, and that Jingqianshu and Jingqianping granules attenuate the abnormal behaviors of model rats. These findings may be useful for the treatment of emotional disorders associated with anger.

## Introduction

In total, ~5% of people worldwide suffer from emotional disorders (1). Among the negative emotions, anger is one of the most intolerable and the most closely associated with the occurrence of diseases, such as cardiovascular events (2). When anger is triggered, the sympathetic nervous system is activated and the sympathoadrenomedullary system is stimulated. As a result, the endocrine system is activated, causing an increase in the levels of cortisol, angiotensin, thyroxine, hyperglycemic factor and hormones from the posterior pituitary lobe in the blood. These exert an effect on the liver and other organs and may thus cause a series of disorders (3). A previous study reported that dysfunction of the network consisting of the nervous, endocrine and immune systems is frequently observed during senescence and other complex diseases, including cancer, diabetes, hypertension and asthma (4). Liu *et al* (5) demonstrated that the levels of plasma adrenocorticotropic hormone and serum serotonin were significantly increased, while the serum level of interleukin-2 was significantly decreased, in the rat anger groups compared with the control group. From population, clinical and animal experimental studies, Qiao *et al* (6) observed that anger was expressed in two different manners: ‘Anger-in’ (tendency to suppress anger) and ‘anger-out’ (tendency to express anger through verbal or physical means); however, the mechanisms underlying this manner of expression remain incompletely understood (6–8).

Emotion-related brain structures include the cerebral cortex, hypothalamus and hippocampus. Among these, the hippocampus plays an important role in context-dependent emotional regulation as a higher-level regulatory center of stress reactions (9). Chronic stress may cause damage to the hippocampal region, leading to changes in its structure and function. The hippocampus is part of the central regulatory system of autonomic nervous activities, and hippocampal damage has been found to be associated with certain cognitive impairments caused by central nervous system diseases (10).

Jingqianping granule is a novel Chinese medicine composed of *Paeonia lactiflora* Pall.*, Cyperus rotundus* Linn., *Fructus Toosendan*, *Bupleurum chinensis* DC., *Rhizoma Chuanxiong*, *Citrus aurantium* L., *Pinellia ternata* (Thunb.), *Alpinia katsumadai hayata*, *Glycyrrhiza uralensis* Fisch., and costus root. The main functional component is saikoside, which has analgesic, sedative, anticonvulsant and antiepileptic effects. A second novel Chinese medicine, Jingqianshu granule, is formulated from *Paeonia lactiflora* Pall., *Angelica sinensis* (Oliv.) Diels., *Bupleurum chinensis* DC., *Atractylodes lancea* (Thunb.) DC., Cortex Moutan and *Cyperus rotundus* Linn. Saikoside A from thorowax roots can significantly reverse the reduction in monoamine neurotransmitters in the brain caused by depression and thus relieve depressive disorders (11). Yang *et al* (12) revealed that the antidepressive effect of thorowax extract may be associated with the strengthening of oxidation resistance in chronic, unpredictable, mild stress rats. It is therefore possible to interfere with emotions using drugs in order to explore potential targets and signaling networks, and thus provide insights into the molecular mechanisms of anger.

In the present study, animal models of anger-in and anger-out emotional reactions were established and the cause and pathogenesis of the condition were systematically investigated via multiple perspectives using DNA chip technology. Furthermore, the effects of the aforementioned Chinese medicines on the anger-in and anger-out animal models were assessed, and molecular-level dynamics were investigated with modern biotechnology from systems biology and bioinformatics.

## Materials and methods

### Reagents

Jingqianping granules were provided by Sichuan Hairong Pharmaceutical Co. Ltd. of the Yangtze River Pharmaceutical Group (Dujiangyan, China). Jingqianshu granules were provided by the Qinhuangdao Shanhaiguan Pharmaceutical Factory (Qinhuangdao, China). Other reagents used included the RNase inhibitor (Toyobo, Osaka, Japan), TRIzol™ (cat no. 15596-026; Invitrogen Life Technologies, Carlsbad, CA, USA) and Moloney Murine Leukemia Virus reverse transcriptase (M-MLV RT; cat no. M1701; Promega Corp., Madison, WI, USA). Primers were synthesized by Jinan BioAsia Biotechnology Co. Ltd (Jinan, China).

### Animals

A total of 50 male Wistar rats (weighing 220–250 g) were provided by the Laboratory Animal Center of Shandong University of Traditional Chinese Medicine (Jinan, China). The Wistar rats were divided into five groups, with 10 rats in each group. The five groups were as follows: Normal control (control), anger-in model (AIM), anger-in Jingqianshu-administered (AIA), anger-out model (AOM) and anger-out Jingqianping-administered (AOA). All animal experiments were conducted in accordance with the ethical guidelines of Shandong University of Traditional Chinese Medicine. The present study was approved by the Institutional Committee for Animal Care and Use of Shandong University of Traditional Chinese Medicine (Approval ID: DWSY200805239).

In the administration period (14 days), the AIA and AOA groups were subjected to intragastric administration of 2.4 g/kg Jingqianshu and 1.6 g/kg Jingqianping granules, respectively, at 09:00 AM daily. The dosages were eight-fold greater the clinical dosages recommended for patients (13). At the same time, the AIM, AOM and control groups were administered an equal volume of sterilized drinking water, which served as the control.

### Establishment of anger-in and anger-out rat models

The anger-in and anger-out rat models were established using the social isolation and resident-intruder methods. For the control group, 10 rats were maintained in standard conditions. A total of 40 rats were used to establish the anger-in and anger-out models. Briefly, for social isolation, 40 rats were kept in separate cages (one rat/cage) for seven days. Subsequently, a resident-intruder test was performed on these 40 rats for a further seven days. Following the resident-intruder test, the aggressive behavior of the rats was observed. Based on the scores of aggressive behavior, the 20 rats with higher scores were defined as the anger-out rat model. The 20 rats with lower scores were defined as the anger-in rat model. The treatments were administered once the models had been established. According to the treatments, the 20 anger-out rats were further divided into the AOA group (n=10, treated with 1.6 g/kg Jingqianping granules) and the AOM group (n=10, treated with equal volume of sterilized drinking water). The 20 anger-in rats were further divided into the AIA group (n=10, treated with 2.4 g/kg Jingqianshu granules) and the AIM group (n=10, treated with equal volume of sterilized drinking water). The 10 rats in the control group were administrated an equal volume of sterilized drinking water. The administration lasted for two weeks. The first day of administration was defined as week 0, the 7th day of administration was defined as week 1, and the 14th day of administration was defined as week 2. At week 0, 1 and 2, the body weight of the rats was recorded. Open-field and aggressive behavior tests were performed at week 0, 1 and 2. After these tests, all rats were sacrificed by cervical dislocation.

### Open-field test

The open-field test was performed as described in our previous study (14). Behavioral changes of the animals in 5 min were recorded by a camera system. A horizontal (crossing) score was recorded as the number of squares that the animal crossed in the time period. A vertical (rearing) score was recorded as the number of times that the animal reared in the same period. The sum of the horizontal and vertical scores was recorded as the total score of each open-field test. The grooming period, grooming times, time sitting in the central square and the number of fecal boli were also recorded.

### Resident-intruder test

The resident-intruder test was subsequently performed as described in our previous study (14). Briefly, two randomly selected rats, which were maintained in separate cages, were placed in the same cage for 15 min. The rats were then moved back to their individual cages. The resident-intruder test was conducted at 12:00 PM daily for seven days.

### Aggressive behavior test

Aggressive behaviors were scored in a blinded manner by three observers who had been identically trained. Each of the recorded videos was examined and the rats’ behaviors were scored by the three observers independently. The scored behavior items included the number of attacks, length of attacking period, number of biting and climbing episodes, length of climbing periods and number of instances of piloerection.

### Gene expression analyses

RNAs were isolated from the control, AIM, AIA, AOM and AOA groups using the Qiagen RNeasy Mini kit (Qiagen, Inc., Valencia, CA, USA), according to the manufacturer’s instructions. Global gene expression levels were analyzed using Affymetrix GeneChip^®^ Expression Analysis (Shanghai Biochip Co., Ltd., Shanghai, China). GeneChip Operating Software version 1.4 (Affymetrix, Santa Clara, CA, USA) was used for the statistical analysis of the data. Probe preparation, microarray hybridization, scanning and data extraction were performed according to the Affymetrix GeneChip Expression Analysis manual instructions. For determination of the differential gene expression, the log ratio was set as ≥0.8 for the genes with upregulated expression and ≤0.8 for the genes with downregulated expression (P<0.5). GeneSpring 10.0 (Agilent Technologies, Inc., Santa Clara, CA, USA) and public databases, including BioCarta, Kyoto Encyclopedia of Genes and Genomes, GenMAPP and National Center for Biotechnology Information Genbank, were searched to analyze the signaling pathways in which the regulated genes were likely to be involved.

### Reverse transcription-quantitative polymerase chain reaction (RT-qPCR)

Total RNA was extracted from the hippocampal tissue of the rats using the Qiagen RNeasy Mini kit, according to the manufacturer’s instructions. RT was conducted according to the instructions of the M-MLV Reverse Transcriptase kit (Promega Corp.). The sequences of the primers are listed in Table I. Following electrophoresis, the gels were subjected to a densitometric scan with the SmartView Image System (SmartView, Irvine, CA, USA) in order to determine the mRNA expression levels of the target genes. The reference gene was β-actin. For amplification of *5-Htr2C*, the following PCR procedure was used: Pre-denaturation at 94°C for 2 min 30 sec, 30 cycles of denaturation at 94°C for 30 sec, annealing at 57.4°C for 40 sec and extension at 72°C for 1 min, and final extension at 72°C for 7 min. For amplification of *GABA_B_R2*, the following PCR procedure was used: Pre-denaturation at 94°C for 2 min 30 sec, 30 cycles of denaturation at 94°C for 30 sec, annealing at 57°C for 40 sec and extension at 72°C for 1 min, and final extension at 72°C for 7 min. For amplification of *5-Htr3B*, the following PCR procedure was used: Pre-denaturation at 94°C for 2 min 30 sec, 35 cycles of denaturation at 94°C for 30 sec, annealing at 53.1°C for 40 sec and extension at 72°C for 1 min, and final extension at 72°C for 7 min. For amplification of β-actin, the following PCR procedure was used: Pre-denaturation at 94°C for 2 min 30 sec, 28 cycles of denaturation at 94°C for 30 sec, annealing at 59°C for 40 sec and extension at 72°C for 1 min, and final extension at 72°C for 7 min. The amplification products were run on agarose gels. The optical density of the PCR products of each gene was analyzed using SmartView^®^ software (Fluke, Everett, WA, USA). The ratio between the optical density of the PCR products of each target gene and the reference gene β-actin was used to determine the relative expression level of the target gene.

### Western blotting

Total proteins were harvested from rat hippocampal tissue, separated on 10% SDS/PAGE gel and subjected to immunoblot analysis. The following primary antibodies were used: Goat anti-rabbit polyclonal γ-aminobutyric acid B receptor 2 (GABA_B_R2; cat. no. G9920; 1:200), which was purchased from Sigma-Aldrich (St. Louis, MO, USA); and goat anti-sheep polyclonal 5-hydroxytryptamine receptor 2C (5-Htr2C; cat. no. sc-1464; 1:200), goat anti-sheep polyclonal 5-Htr3B (cat. no. sc-51198; 1:200) and mouse anti-chicken monoclonal β-actin (cat. no. sc-47778; 1:10,000), which were all obtained from Santa Cruz Biotechnology, Inc., (Dallas, TX, USA). The secondary antibodies used were donkey anti-goat immunoglobulin G (IgG)-horseradish peroxidase (HRP) (cat. no. sc-2020; 1:5,000; Santa Cruz Biotechnology, Inc.) and goat anti-mouse IgG-HRP (cat. no. sc-2005; 1:10,000; Santa Cruz Biotechnology, Inc.). Bound antibodies were detected using an ECL system (Pierce Biotechnology; Thermo Fisher Scientific Inc., Rockford, IL USA). The immunoblot experiments were repeated ≥3 times. The mean normalized optical density of the target protein band relative to that of the β-actin band from the same individual was calculated.

### Statistical analysis

All values are expressed as the mean ± standard deviation. Data were analyzed with SPSS 16.0 statistical analysis software (SPSS., Inc., Chicago, IL, USA). Two-way analysis of variance was used to compare the mean values from multiple groups of samples. Fisher’s Least Significant Difference t-test was used for comparisons between two groups. The significance level α was set at 0.05. P<0.05 was considered to indicate a statistically significant difference.

## Results

### Result of the rat behavioral tests

**Figure 1:**
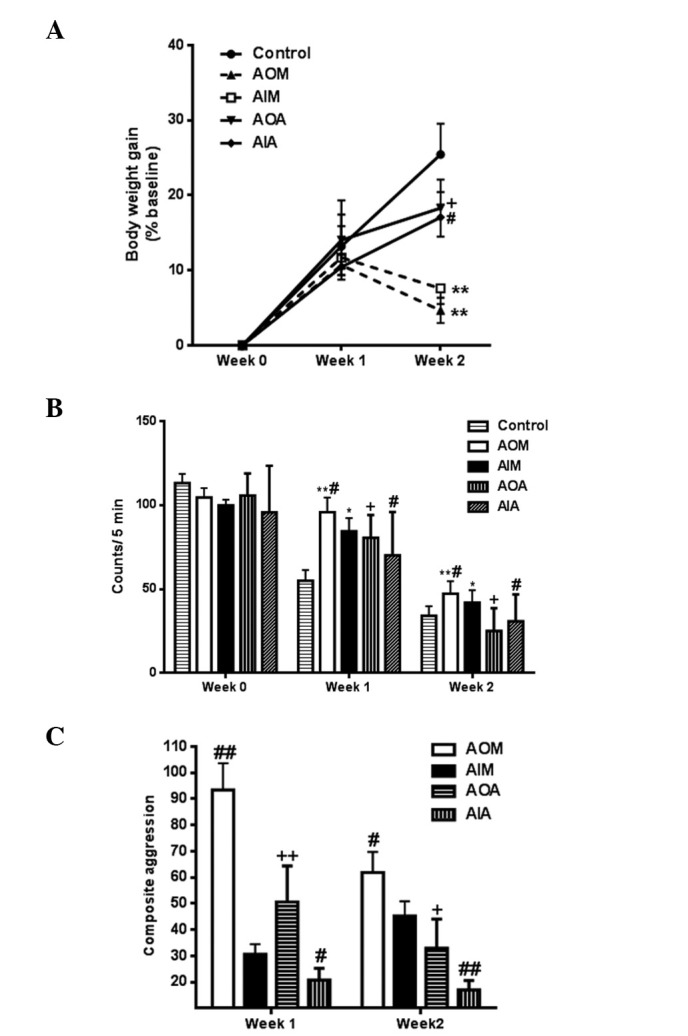
Tests using the rat model. (A) Comparison between the weights of the rats prior to and following model establishment. (B) Comparison of the open-field test scores of different groups. (C) Comparison of the aggression scores of the rats in the different groups. AIM, anger-in model; AOM, anger-out model.^*^P<0.05 and ^**^P<0.01, as compared with the control group; ^#^P<0.05 and ^##^P<0.01, as compared with the AIM group. ^+^P<0.05 and ^++^P<0.01, as compared with the AOM group.

To investigate the genes associated with the anger-in and anger-out emotions in humans, a rat model was established. A total of 50 male Wistar rats were divided into five groups (control, AIM, AIA, AOM and AOA), with 10 rats in each group. Following the establishment of the rat models, the weights of the rats were measured over the first 2 weeks. As shown in Fig. 1A, no significant differences in body weight were observed among the rat groups in the first week; however, after 2 weeks, the body weights of the rats in the AIM and AOM groups were significantly decreased compared with those of the control group rats. The body weight of the rats in the AIA group at week 2 was significantly higher, as compared with the AIM group (P<0.05). In addition, at week 2, the body weight of the rats in the AOA group was also significantly higher, as compared with the AOM group (P<0.05).

Open-field tests were performed to assess the behavioral changes in the rats. As indicated in Fig. 1B, the scores of the rats in the AIM and AOM groups were significantly higher (P<0.05) than those in the control group in the first and second weeks. In the first and second weeks, the scores of the AIM group were significantly lower, as compared with the AOM group (P<0.05), whereas the scores were significantly higher, as compared with the AIA group (P<0.05). Furthermore, the AOA group had significantly lower scores in the first and second weeks, as compared with the AOM group (P<0.05).

As shown in Fig. 1C, the aggression scores of the rats in the AOM group were significantly higher than those of the AIM group rats in the first and second weeks. In addition, the AIA group had significantly lower aggression scores in the first and second weeks, as compared with the AIM group (P<0.05). Furthermore, in the first and second weeks the AOA groups had significantly lower aggression scores, as compared with the AOM group (P<0.05).

### Differentially expressed genes in the rat groups

To investigate the gene expression profiles in the control, AIM, AIA, AOM and AOA groups, RNA was extracted from each animal and subjected to global gene expression analysis. For the determination of differential gene expression, the log ratio was set as ≥0.8 for the genes with upregulated expression and ≤0.8 for the genes with downregulated expression (P<0.5).

The number of regulated genes in each group is shown in Table II. The numbers in the control group were set as 0. The expression of 63 genes was upregulated and that of 153 genes was downregulated in the AIM group. In the AIA group, the expression of 23 genes was upregulated and that of 196 genes was downregulated. In the AOM group, the expression of 246 genes was upregulated and that of 256 genes was downregulated. In the AOA group, the expression of 174 genes was upregulated and that of 165 genes was downregulated. The variation in gene regulation may have resulted from the differential levels of stimulation in these groups. The drug-administered groups (AIA and AOA) showed decreased numbers of up- and downregulated genes as compared with the model groups (AIM and AOM), respectively, suggesting that Jingqianshu and Jingqianping granules may attenuate the behavioral changes of the rats.

### Analysis of the 5-Htr2C, GABA_B_R2 and 5-Htr3B mRNA levels

**Figure 2:**
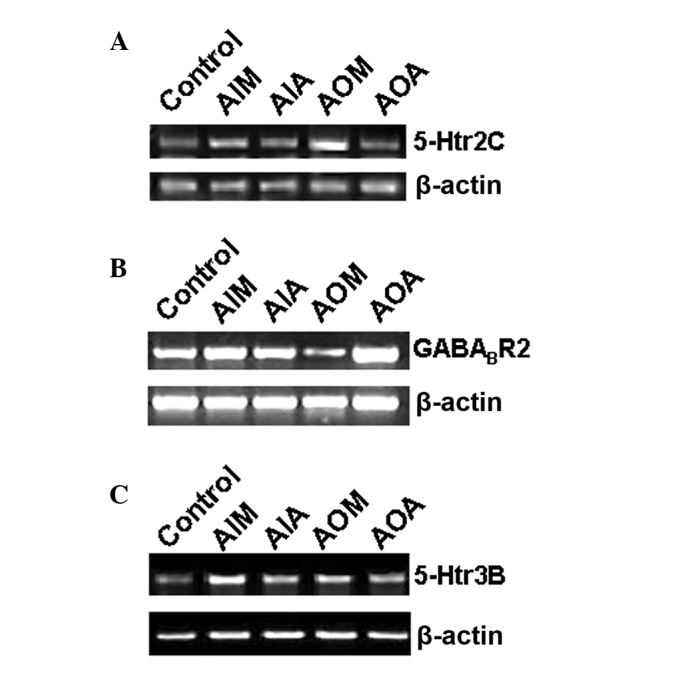
Reverse transcription-quantitative polymerase chain reaction results for the (A) *5*-*Htr2C*, (B) *GABA_B_R2*, and (C) *5*-*Htr3B*. genes Total RNA was extracted from the hippocampal tissue of the rats and reverse transcription was performed. Following electrophoresis, the gels were subjected to a densitometric scan with a SmartView Image System (Smartview, Irvine, CA, USA) in order to determine the mRNA expression levels of the target genes and the reference gene, β-actin. AIM, anger-in model; AIA, anger-in Jingqianshu-administered; AOM, anger-out model; AOA, anger-out Jingqianping-administered.

Previous studies have reported that the genes *5*-*Htr2C* and *GABA_B_R2* are associated with irritability and that *5*-*Htr3B* is associated with depression (15–17). DNA chip analysis in the present study revealed that the three genes were expressed differentially in the AIM and AOM rat groups. The three genes were then subject to RT-qPCR analysis; the RT-qPCR results (Fig. 2) were consistent with the findings of the DNA chip analysis (Table II). As shown in Fig. 2A, the relative level of *5*-*Htr2C* mRNA in the hippocampal tissue of the rats in the AIM and AOM groups was higher than that in the control group (P<0.01). The drugs attenuated the increase in the level of *5*-*Htr2C* mRNA, as indicated by comparisons of the levels of mRNA in the AIA and AOA groups versus those in the AIM and AOM groups. As shown in Fig. 2B, the relative mRNA level of *GABA_B_R2* was upregulated in the AIM group but downregulated in the AOM group (P<0.01). The drugs attenuated the alterations in the *GABA_B_R2* mRNA expression, as indicated by comparisons of the levels of mRNA in the AIA and AOA groups versus those in the AIM and AOM groups. As shown in Fig. 2C, the relative levels of *5-Htr3B* in the AIM and AOM groups were higher than those in the control group (P<0.01). The drugs attenuated the increase in *5*-*Htr3B* mRNA, as indicated by comparisons of the levels of mRNA in the AIA and AOA groups versus those in the AIM and AOM groups. These results therefore suggest that Jingqianshu and Jingqianping granules attenuated the alterations in the levels of mRNA induced by anger-in and anger-out emotions.

### Protein levels of target genes revealed by western blot analysis

**Figure 3:**
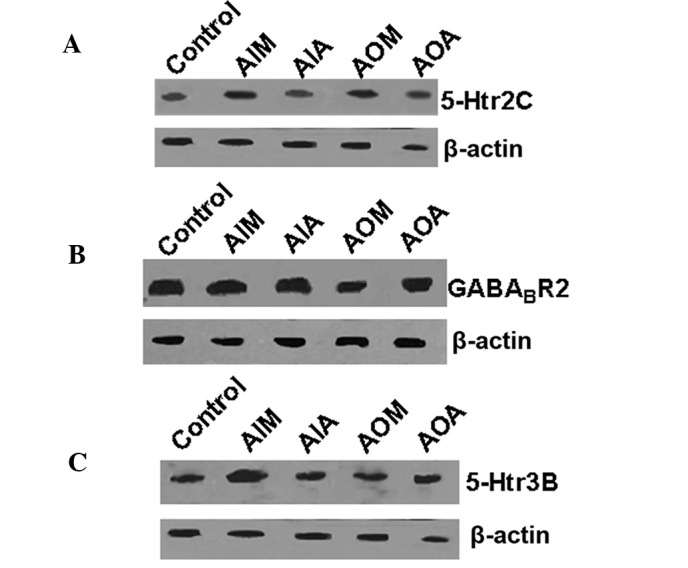
Western blotting results of the (A) 5-Htr2C, (B) GABA_B_R2, and (C) 5-Htr3B proteins in the rat hippocampal tissue. Total proteins were harvested from rat hippocampal tissue, separated on 10% SDS/PAGE gel and subjected to immunoblot analysis. Bound antibodies were detected using an ECL system. β-actin was the reference protein and the immunoblot experiments were repeated ≥3 times. AIM, anger-in model; AIA, anger-in Jingqianshu-administered; AOM, anger-out model; AOA, anger-out Jingqianping-administered.

To further determine the expression profiles of the *5*-*Htr2C*, *GABA_B_R2*, and *5*-*Htr3B* genes, the levels of the proteins encoded by the three genes were examined by western blotting. As shown in Fig. 3A, the relative levels of the 5-Htr2C protein in the hippocampal tissue of rats in the AIM and AOM groups were higher than those in the control group rats (P<0.01). The drugs attenuated the increase in 5-Htr2C protein expression, as indicated by comparisons of the levels of protein in the AIA and AOA groups versus those in the AIM and AOM groups.

As shown in Fig. 3B, the relative level of GABA_B_R2 protein was upregulated in the AIM group but downregulated in the AOM group (P<0.01). The drugs attenuated the alterations in GABA_B_R2 protein expression, as indicated by comparisons of the levels of protein in the AIA and AOA groups versus those in the AIM and AOM groups.

As shown in Fig. 3C, the relative level of 5-Htr3B protein in the AIM and AOM groups was higher than that in the control group (P<0.01). The drugs attenuated the increase in 5-Htr3B protein expression, as indicated by comparisons of the levels of protein in the AIA and AOA groups versus those in the AIM and AOM groups. In combination, these results suggest that Jingqianshu and Jingqianping attenuated the protein expression changes induced by anger-in and anger-out emotions.

## Discussion

In the present study, anger emotion rat models were established through social isolation and resident-intruder methods. Aggressive behavior and open-field tests were carried out to assess the emotional status of the rats. It was found that the scores of the model rats were significantly higher than those of the rats in the control group (P<0.05). The results suggested that the models were well established and that the combination of open-field and composite aggressive behavioral scores may be used to distinguish anger-in and anger-out model rats from one other and from normal rats at the initial stages of anger presentation. The behavioral changes in the anger-in and anger-out model rats may resemble those in human patients, which may aid with clinical application and treatment options.

In the current study, a number of differentially expressed genes were revealed to be representative of anger-in and/or anger-out emotions. Preliminarily comparisons at the molecular level have revealed the genetic mechanisms that may trigger anger-in and anger-out emotions. Furthermore, the results of the present study have demonstrated the main target genes and signals involved in the regulatory pathways of Jingqianshu and Jingqianping granules during their intervention against anger-in and anger-out emotions.

In the treatment of the anger-out emotion, Jingqianping can modulate the expression of important regulatory factors, such as adenylate cyclase (AC) and JNK/SAPK-inhibitory kinase, and thus affect upstream pathways, such as ligand-receptor interaction in neuron activation and the AC/cyclic adenosine monophosphate-dependent protein kinase pathways (18). The effect of Jingqianping can then be transmitted via downstream signaling pathways, including mitogen-activated protein kinase (MAPK), Ca^2+^ signal transduction and gonadotropin-releasing hormone, to be activated or inhibited. As a result, the overexcitation of the hypothalamic-pituitary-adrenal axis and hypofunction of the hypothalamic-pituitary-thyroid and hypothalamic-pituitary-gonadal axes are counterbalanced, and the dysfunction in the network comprising the nervous, endocrine and immune systems is relieved (19–21).

The main targets of Jingqianshu in its effect on anger-in emotion include *PRLR*, *GRM8*, *FGFR2*, *HCRT*, *PLA2G5*, *BACE1* and *NCR3* (22). The biological functions of these targets are associated with the protection of neurons, inhibition of apoptosis, protection of signal transduction through synaptic transmission, stabilization of the Ca^2+^ pathway and antagonism of anoxia. Compared with the regulatory function of Jingqianping granules, fewer target genes are regulated by Jingqianshu (22). With regard to the functional mechanisms of Jingqianshu, the relevant ligand-receptor interactions involved in neuron activation and other signaling pathways, such as glycerophospholipid metabolism, MAPK and natural killer cell-mediated cytotoxicity, require further study.

Compound prescriptions in Traditional Chinese Medicine have the advantage of integrated regulatory functions through multiple ingredients, links and targets in the treatment of chronic, complex and multifactorial disorders. The chemical complexity of the compound prescriptions and the diversity of their formulae and functions have, however, made them difficult to be determined, which has hindered the international application of Chinese medicine. With modern biological technology, it has been revealed that Realgar-Indigo naturalis formula can be used for the treatment of acute promyelocytic leukemia through multiple-target and synergistic mechanisms (23). This is, to the best of our knowledge, the first time that a Chinese compound prescription mechanism has been elucidated at the level of the molecular networks.

In conclusion, the present study has investigated how anger emotions cause disease and evaluated the efficacy of Chinese medicines for the treatment of anger emotions. Rat models of anger emotions were established, and human genomic and proteomic databases were searched. Using the rat models, the preliminary application of Jingqianshu and Jingqianping and the functional mechanisms underlying their effect on anger emotions were investigated at the level of regulatory molecular networks.

## Figures and Tables

**Table I:** Primers used in the present study.

Genes	Sequences	Product size (bp)
β-actin	F: 5′-TGGTGGGTATGGGTCAGAAGGACTC-3′ R: 5′-CATGGCTGGGGTGTTGAAGGTCTCA-3′	265
*5-Htr2C*	F: 5′-TTCGTTCTCATCGGGTCCTT-3′ R: 5′-CACATAGCCAATCCACACAA-3′	441
*GABA_B_R2*	F: 5′-CCATCTGGCTTGGCATTGTC-3′ R: 5′-CTGTGCTCTCTGTGAAGTTGC-3′	370
*5-Htr3B*	F: 5′-TCTCTCCCT CTCAGTGCCAT-3′ R: 5′-CAAGAGGCTCACAACATAGGC-3′	582

F, forward; R, reverse.

**Table II:** Screening results of differentially expressed genes among the groups.

Groups	Upregulated genes (n)	Downregulated genes (n)
Control	0	0
AIM	63	153
AIA	23	196
AOM	246	256
AOA	174	165

AIM, anger-in model; AIA, anger-in Jingqianshu-administered; AOM, anger-out model; AOA, anger-out Jingqianping-administered.

## References

[1] Kessler RC, Aguilar-Gaxiola S, Alonso J (2009). The global burden of mental disorders: an update from the WHO World Mental Health (WMH) surveys. Epidemiol Psichiatr Soc.

[2] Mostofsky E, Penner EA, Mittleman MA (2014). Outbursts of anger as a trigger of acute cardiovascular events: a systematic review and meta-analysis. Eur Heart J.

[3] Tao HY, Qiao MQ, Wang WY (2009). The dependability of anger pathopoiesis and psychological stress. Zhejiang Zhong Yi Yao Da Xue Xue Bao.

[4] Wang CX, Zheng HX, Wang JW (1997). A probing into the imbalance of immunity system, endocrine, nerves and the liver affected by anger. Liaoning Zhong Yi Za Zhi.

[5] Liu XW, Qu HD, Zhang HM (2004). Content change of ATCH/CORT/IL-2/IL-8 in blood from rats with Qi impaired by anger. Zhong Guo Quan Ke Yi Xue.

[6] Qiao MQ, Wang WY, Zhang HY (2007). Epidemiological survey on etiology of Gan-qi inversion syndrome and Gan-qi stagnation syndrome and study on the evocative mode of emotional diseases. Zhongguo Zhong Xi Yi Jie He Za Zhi.

[7] Chao YB, Wei S, Qiao MQ, Wang JQ, Zhang HY (2010). Analysis of monoamine neurotrasmitter content in serum and different encephalic regions of PMS liver-qi invasion, depression rat models. Yi Xue Yan Jiu Za Zhi.

[8] Qiao MQ, Zhang HY, Wang HJ (2006). Relationship between anger-in and anger-out and premenstrual syndrome, Gan-qi inversion syndrome and Gan-qi stagnation syndrome. Shanxi Yi Yao Za Zhi.

[9] Czéh B, Michaelis T, Watanabe T (2001). Stress-induced changes in cerebral metabolites, hippocampal volume, and cell proliferation are prevented by antidepressant treatment with tianeptine. Proc Natl Acad Sci USA.

[10] Zuo HY, Wang DW (2007). Advances of hippocampus proteomics. Zhong Guo Lin Chuang Jie Pou Xue Za Zhi.

[11] Ge HY, Chen B, Xu D (2008). Influence of Saikosaponina A on monoamine neurotransmitters and the corresponding metabolin compositions in depressed rats’ brain. Zhong Guo Gao Deng Xue Xiao Hua Xue Xue Bao.

[12] Yang XY, Ma SP, Qu R (2007). Effects of Bupleurum chinense extracts on lipid peroxidation in chronic unpredictable mild stress model of depression in rats and lymphocyte proliferation in mice. Zhong Guo Yao Ke Da Xue.

[13] Xu SY, Bian RL, Chen X (2002). Methodology of Pharmacological Experiment.

[14] Wei S, Zhang H, Gao J (2010). Impact of social isolation and resident intruder stress on aggressive behavior in the male rat. Neural Regen Res.

[15] Rauser L, Savage JE, Meltzer HY, Roth BL (2001). Inverse agonist actions of typical and atypical antipsychotic drugs at the human 5-hydroxytryptamine(2C) receptor. J Pharmacol Exp Ther.

[16] Ulrich D, Bettler B (2007). GABA(B) receptors: synaptic functions and mechanisms of diversity. Curr Opin Neurobiol.

[17] Blier P (2003). The pharmacology of putative early-onset antidepressant strategies. Eur Neuropsychopharmacol.

[18] Guo YH, Sheng Wei, Gao J, Song CH, Qiao MQ (2012). Effect of Jingqianping granule on gene expression profile in the hippocampus of the anger-out rat model. Zhong Guo Yao Li Xue Tong Bao.

[19] Páez-Pereda M (2005). New drug targets in the signaling pathways activated by antidepressants. Prog Neuropsychopharmacol Biol Psychiatry.

[20] Wang H, Zhang M (2012). The role of Ca^2+^-stimulated adenylyl cyclases in bidirectional synaptic plasticity and brain function. Rev Neurosci.

[21] Ganea D, Rodriguez R, Delgado M (2003). Vasoactive intestinal peptide and pituitary adenylate cyclase-activating polypeptide: players in innate and adaptive immunity. Cell Mol Biol (Noisy-le-grand).

[22] Guo YH, Gao J, Xu KY, Song CH, Qiao MQ (2011). Effect of Jingqianshu granule on gene expression profile in the hippocampal of the anger-in rat model. Zhong Guo Yao Li Xue Tong Bao.

[23] Wang L, Zhou GB, Liu P (2008). Dissection of mechanisms of Chinese medicinal formula Realgar-Indigo naturalis as an effective treatment for promyelocytic leukemia. Proc Natl Acad Sci USA.

